# Counteranion Effects on the Incorporation of Photosystem I with Poly(3,4-ethylenedioxythiophene) (PEDOT)

**DOI:** 10.1021/acsomega.4c09448

**Published:** 2025-03-07

**Authors:** William R. Lowery, G. Kane Jennings, David E. Cliffel

**Affiliations:** † Department of Chemistry, Vanderbilt University, Nashville, Tennessee 37235-1822, United States; ‡ Department of Chemical and Biomolecular Engineering, Vanderbilt University, Nashville, Tennessee 37235-1604, United States

## Abstract

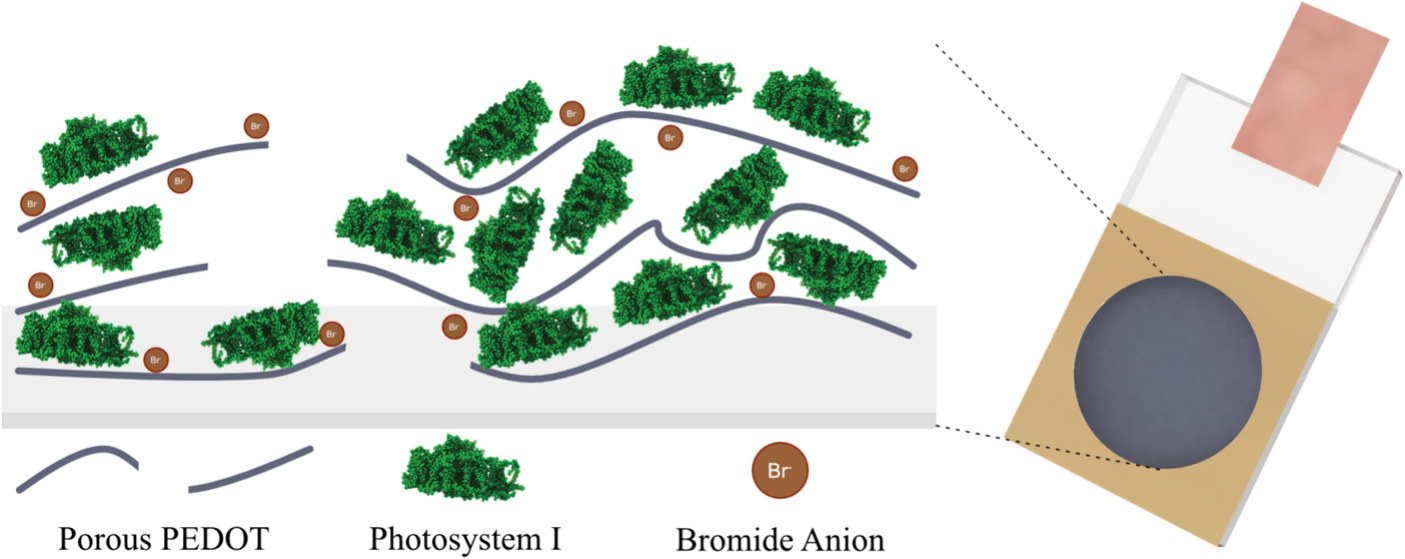

Conductive polymers provide an effective interface for proteins, particularly in photovoltaic applications. The synthetic toolbox affords a variety of options in which to fine-tune protein–polymer properties toward better engineered materials. While prior work has shown compatibility between Poly(3,4-ethylenedioxythiophene) (PEDOT) and Photosystem I, a detailed study of counteranion effects with interfacing Photosystem I has yet to be performed. This study, which fills a significant gap in the field, involves the synthesis of PEDOT films with a variety of different potassium-based salts. Morphology, capacitance, and impedances all varied across the films when deposited in the presence of the different anions. These properties were evaluated independently before interfacing PSI. After incorporating PSI within these films through entrapment and deposition approaches, the counteranion dependent properties were explored further through the photoactivity of these composite films. Results showed that films produced with the bromide anion provided the highest photocurrent output due to the porous leaf-like structure of the conducting polymer matrix.

## Introduction

Nature has the amazing ability to complete a vast array of complex chemical processes. Of particular note is the ability of plants to efficiently harvest sunlight and transform it into storable chemical energy. Over millions of years of evolution, plants and certain bacteria have designed specific proteins to accomplish the task of photosynthesis.^1^ One of these remarkable proteins, Photosystem I provides an electron of sufficiently high reducing potential to ultimately drive ATP generation.^2^ This ATP is the basis of chemical energy for biological organisms. Through photosynthesis, nature has provided great inspiration for humans to adapt their light-harvesting approaches and even to incorporate some of nature’s aspects into their designs.

Photosystem I is a relatively stable membrane protein that is capable of functioning outside its native cellular environment.^3^ Along with its stability, PSI effectively excites electrons due to its near-perfect internal quantum efficiency.^4,5^ PSI is comprised of two reaction centers: the P700 site, where the electron is introduced to the protein and F_b_ where oxidized species may utilize the excited electron.^4^ The potential difference between these two sites, only ∼10 nm apart, is slightly higher than 1 V, providing the F_b_ site with the largest reducing potential in nature.^5,6^ This high potential along with its *ex vivo* stability offers great promise for PSI to accomplish a wide variety of light-driven reduction processes.^7^ Effectively utilizing this potential that PSI generates remains one of the most critical tasks in the realm of PSI research. Accessing the reaction sites of PSI through use of electron mediators has helped greatly in this effort.^8,9^ Three dimensional nanomaterial approaches have also shown great promise, particularly conductive polymers by providing unique, high surface area interfaces for PSI.^5,10^ Conductive polymers tend to be primarily organic with some exhibiting inorganic components.^11^ These fully organic polymers conduct electrons in a chain of conjugated double bonds.^12^ Of the many conductive polymers available, poly(3,4-ethylenedioxythiophene) polystyrenesulfonate (PEDOT:PSS) is arguably one of the most popular due to its synthetic customizability, aqueous processability, and biocompatibility.^13^

Photosystem I and conductive polymers are compatible in a variety of settings and can be interfaced in a number of ways. In one case, composite polymer/protein photoactive films were produced through a single electropolymerization step by entrapping PSI within a growing polymer of aniline.^14^ In another, conductive polymers were initiated and grown throughout a multilayer film of PSI by introducing the monomer in the vapor phase.^15^ Viologen based conductive polymers have also been shown to be capable of effectively interfacing with PSI in both layer-by-layer assemblies and solid state device fabrications.^16,17^ Additionally, osmium-based redox polymers have been used as an effective interface for both PSI and PSII.^18−20^ In one study, a hydrogel redox polymer was utilized to not only accept electrons from PSII, but also immobilize the protein to the electrode.^18^ A similar approach was taken to immobilize PSI to study the effects of oxygen concentration on the long-term stability of these PSI bioelectrodes.^20^ PEDOT and PSI have specifically been shown to be compatible in a variety of approaches, such as by a layer-by-layer assembly methodology and more recently, in a simultaneous spin coating of the two materials.^21,22^ In both cases, these approaches demonstrated hybrid films that showcased the synergistic effects that conductive polymer and PSI composites can exhibit.

Despite a growing body of work detailing how to interface PSI within a conductive polymer network, the effect of counteranions has yet to be explored. This interest in counteranions is well founded in previous work with conductive polymers, demonstrating how various properties, such as morphology, absorption, and capacitance, can be affected.^23−29^ Spanninga et al. used X-ray photoelectron spectroscopy (XPS) to show that PEDOT does exhibit a greater affinity for certain anions, especially bromide.^26^ Another team led by Yanagida reported that while PEDOT:PSS was the only commercially available form of PEDOT, different morphologies were obtainable by altering the anion in the synthesis process.^27^ The same group later reported how PEDOT polymerized in the presence of larger anions exhibited lower impedances.^28^ Additionally, the capacitances and electrochromic properties of PEDOT were also reported to be dependent on the counteranion of synthesis.^29^ Seeking to fill a gap in the literature, we present here a study on the effects of counteranions on both the incorporation of PSI with the popular PEDOT polymer, as well as the photoelectrochemical properties of the composite film.

## Experimental Section

### Photosystem I Extraction

Photosystem I was isolated, quantified, and stored according to procedures described by Baba et al.^30^ A more thorough description of the extraction procedure was published more recently, which utilized a slightly modified version of buffers and thylakoid lysing procedure.^3^ In brief, spinach leaves were blended and centrifuged to isolate the thylakoids. After lysing the membranes to liberate the photosystems, an ionic exchange column was utilized to isolate PSI from the rest of the cell debris. The protein was last solubilized by Triton X-100 and stored at −80 °C in elution buffer at a concentration of ∼2 mg/mL as determined by Baba’s assay.^30^ Before use in certain deposition cases, the protein was dialyzed with a 10 kDa MWCO dialysis membrane in a 1:1000 by volume setup to remove Triton X-100 surfactant to aid the deposition of PSI within the conducting polymer film.

### Preparation of PEDOT Films

Monomer stock was made from 3,4-ethylenedioxythiophene (EDOT) 97% water suspension and diluted to a concentration of 0.01 M EDOT and 0.1 M KX where X represents the anion of interest. Potassium-based salts were used exclusively due to a previous study reporting the accompanying anion to potassium salts as showing the strongest interactions with PEDOT, providing an excellent system to probe the effects of varying the counteranion.^31^ For polymerization solutions, the stock of EDOT/KX was combined with either a PSI or control elution buffer in a 4:1 volume ratio. Chloride, bromide, iodide, nitrate, nitrite, perchlorate, chlorate, and triflate were all tested as potential anion candidates. Of these, only chloride, bromide, perchlorate, and nitrate produced films with sufficient quality for further testing. Of this set, films deposited in the presence of perchlorate did not exhibit enough adherence to the underlying substrate for complete testing. As such, some data sets have a reduced number of perchlorate samples due to their mechanical instability.

PEDOT films were electrodeposited onto indium tin oxide (ITO)-coated glass slides. Pretreatment of these ITO slides included sonication in acetone, ethanol (180 proof), and water for 5 min each cycle. Afterward, these substrates were covered with electrochemical sample masks (Gamry) to restrict the electroactive surface area to 0.316 cm^2^. Finally, copper tape was attached to the top of the slide to allow for better connection to the alligator clips of the potentiostat.

To focus variability on the counteranions themselves, potentiostatic techniques were utilized for electropolymerization. Specifically, amperometric i-t was used to apply a fixed potential of 1.6 V to the working ITO electrode for 400 s. A strong anodic current dominated initially until a diffusion-limited plateau was quickly reached. During this period, EDOT monomer was oxidized to a radical form where it could either nucleate on the substrate, further an already growing polymer chain, or terminate with another radical.^32^

### Incorporation of Photosystem I

Photosystem I was incorporated into the PEDOT material through various approaches. Entrapment involved introducing PSI into the system during electropolymerization. Here, a mixture of 1:4 parts of PSI to EDOT stock solutions was electropolymerized to yield composite films with PSI expected to be spread throughout the interior of the film. In another approach, the films were produced in the absence of PSI as previously described. Afterward 50 μL of dialyzed PSI solution was then dropcast onto the film and dried under vacuum to create a PSI multilayer atop the PEDOT film, concentrating the protein atop the film. In the third approach, the two previous strategies were combined to provide both a high concentration of protein atop the film along with spreading some PSI throughout the interior of the film. The composite films were produced, and then, a PSI multilayer was introduced via drop casting followed by drying under vacuum. Support for PSI incorporation was obtained using UV–vis and FTIR spectroscopies and are presented in SI1 and SI2 respectively.

### Electrochemical Instrumentation

All electrochemical measurements were performed using a CHI660A electrochemical workstation along with a Pt mesh counter electrode and Ag/AgCl reference electrode. A 0.1 M KCl electrolyte was utilized in all experiments with the exception of PEDOT syntheses. Electrochemical impedance spectroscopy (EIS) measurements were taken similarly to the above setup at a bias DC voltage of the PEDOT film’s LUMO under study (as determined via CV) to provide a mixed valence state of the polymer with an AC perturbation of 5 mV and a frequency range of 10 mHz to 10 kHz.

### Photoactivity Measurements

The photoactivities of the films were tested with photochronoamperometry (PCA). In this technique, the system was held at the open circuit potential (OCP) in the absence of any illumination, which is the potential needed to prevent any appreciable current from flowing between the working and counter electrodes. A redox pair of 2,6-dichlorophenolindophenol (DCPIP) and sodium ascorbate (NaAsc) was used to mediate electron transfer between the reaction centers of PSI and the electrode. This mediator was chosen based upon previously reported success.^33^ In this system, NaAsc serves as a sacrificial electron donor to reduce DCPIP to DCPIPH_2_, which will in turn reduce the P_700_ site of PSI. Upon reduction of P_700_, DCPIP may then be rereduced by the working electrode to produce a measurable current. This cycle repeats as photons drive the electron from P_700_ to F_b_, where it reduces dissolved oxygen.

### Morphological Characterizations

All scanning electron microscopy was performed on a Zeiss Merlin scanning electron microscope equipped with an Everhart-Thornley detector. Images were taken at a working distance around 5 mm, an accelerating voltage of 2 kV, and a beam current of 100 pA

## Results and Discussions

**Figure 1:**
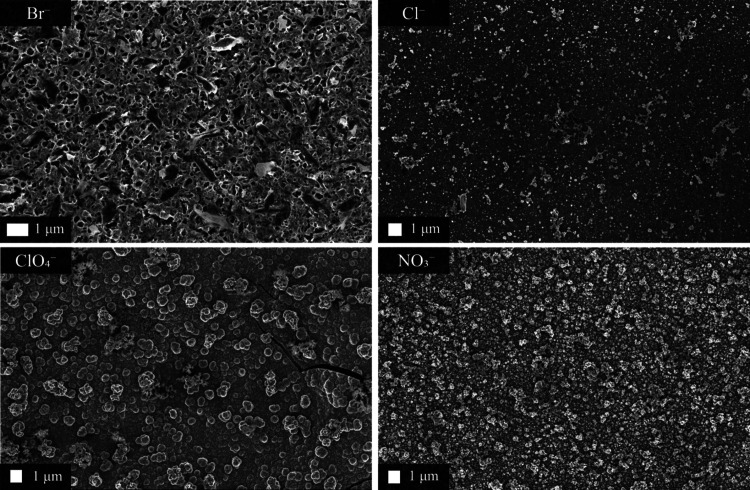
Scanning electron micrographs of PEDOT films polymerized in the presence of KBr, KCl, KClO_4_, and KNO_3_. The morphologies have been termed: porous leaf-like, nonuniform, cracked globular, and globular, respectively.

Following synthesis with the various counteranions, PEDOT films were characterized with a variety of techniques to determine how properties had been affected by the electrolytes used. Scanning electron microscopy was employed to showcase their differences in morphology. As shown in Figure 1, PEDOT films exhibit a unique morphology with each anion, supporting the assertion that counteranions play a large role in electropolymerization of EDOT. The PEDOT film polymerized in the presence of KBr exhibited a highly porous morphology consistent with a porous leaf, given the porosity and leaf-like blades that can be seen. PEDOT films grown in nitrate and perchlorate exhibited the commonly encountered globular structure, while the crack shown in the perchlorate panel suggests poor mechanical stability of the film and incompatibility with the substrate.^27,29,34^ Finally, the PEDOT film grown in chloride exhibited a low, nonuniform coverage, with greatly reduced material deposited, as compared to that of PEDOT films grown with the other anions. These features were also observed in films polymerized in the presence of PSI and are presented in SI3 along with a feature size summary in SI4.

**Figure 2:**
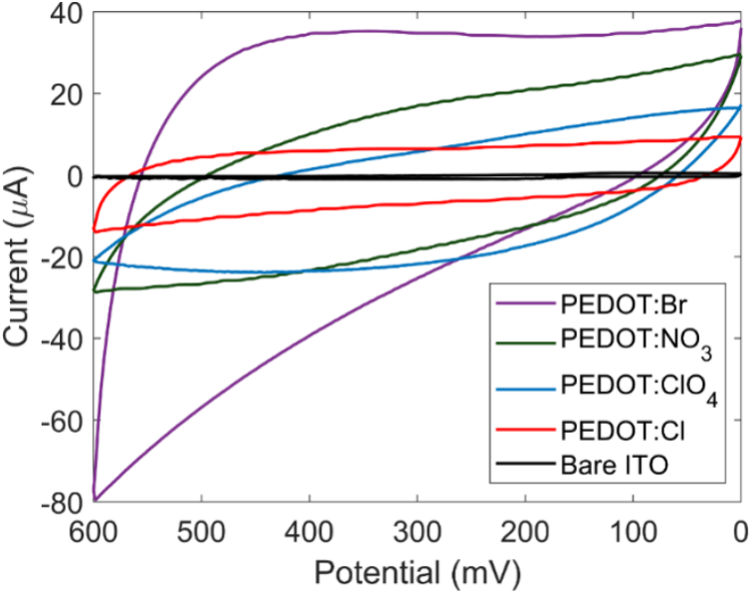
Cyclic voltammogram of PEDOT films deposited on ITO working electrodes in 100 mM KCl at a scan rate of 100 mV/s. Potentials are reported relative to a Ag/AgCl reference electrode.

Following SEM characterization, cyclic voltammetry (CV) was used to determine the range of surface areas from the different morphologies. Consistent with its porous morphology, the PEDOT film grown in KBr exhibited the greatest electroactive surface area, as quantified by measuring the capacitances and comparing the non-Faradaic current. Figure 2 illustrates how the capacitances of each film compare while the accompanying table includes values for the charging currents. Charging current (*i*_c_) can be represented by
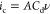
1where *A* is the surface area of the electrode, *C*_d_ is the double layer capacitance, and ν is the scan rate. As the scan rate is consistent across the different films, the charging current will scale with the surface area and double layer capacitance factors. The bare ITO sample serves as the baseline with PEDOT films showing larger charging currents, suggesting that the films exhibit greater electroactive surface area. The greatest charging current was exhibited by PEDOT: Br as expected due to the porosity observed in the above SEM images as summarized by Table 1.

**Table 1:** Non-Faradaic Charging Currents of PEDOT Films Showcasing a Boost in Surface Area Recorded at 100 mV

sample	charging current (μA)
Bare ITO	0.5
PEDOT: Cl	8.2
PEDOT: ClO_4_	14.0
PEDOT: NO_3_	24.4
PEDOT: Br	34.7

This drastically different morphology that is exhibited by the bromide film is explained in detail in SI5. Briefly, it is hypothesized that the oxidation of bromide to bromine occurs alongside the oxidative polymerization of EDOT generating a local blocking layer for further PEDOT deposition and thus, leads to a porous structure.

**Figure 3:**
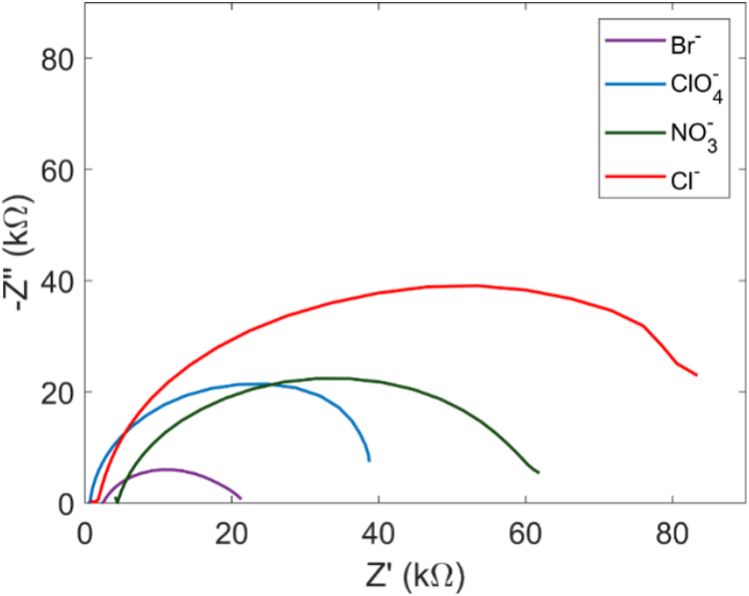
Nyquist plots of PEDOT films taken at the bias potential of the E_1/2_ of the film in 100 mM KCl. PEDOT:Br showcases the least resistance to charge transfer as indicated by the smallest semicircle.

A deeper examination into the electrochemical properties of the PEDOT films was achieved via electrochemical impedance spectroscopy (EIS), and the resulting Nyquist plots are shown in Figure 3. Here, the redox activity of the PEDOT film was characterized through transfer of electrons into and out of the polymer’s LUMO. The below overlay shows how well the polymer films modified the original ITO substrate. The smallest semicircle associated with PEDOT:Br showcases the ease with which electrons are cycled through the film as opposed to the less porous films synthesized with other electrolytes.

These findings of improved performance in PEDOT: Br films are best explained through adoption of the ion movement model, described elsewhere.^11,35,36^^37^ Briefly, anions such as chloride that are bound more tightly to the PEDOT backbone prevent charge mobility whereas polyatomic anions are too bulky for fluid movement. Bromide provides an excellent middle ground of charge polarizability along with size for movement, in addition to the porosity of the film itself.

**Figure 4:**
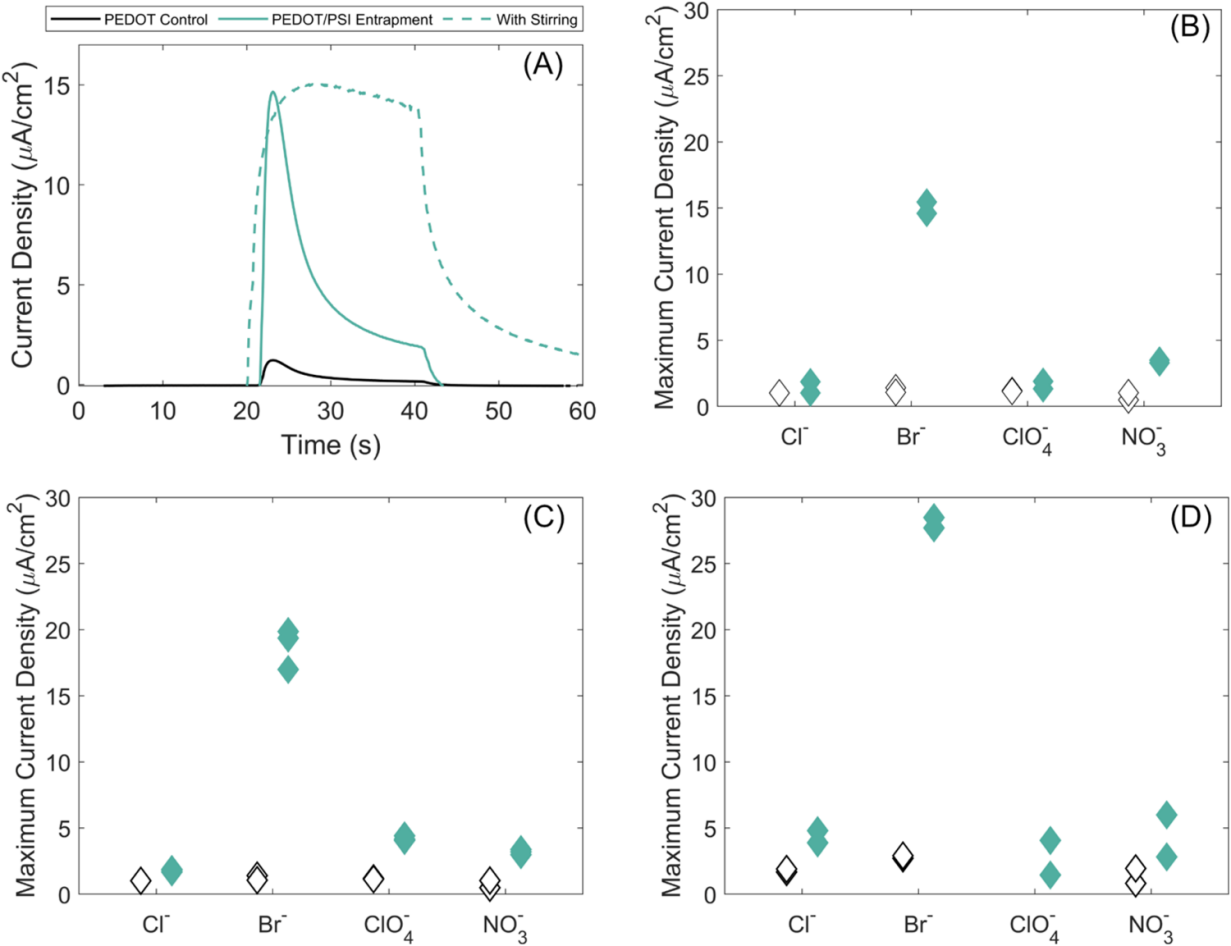
Photochronoamperometry (PCA) measurements of PEDOT/PSI composite films where green points represent PSI containing films and hollow points represent controls. (A) Example PCA measurements of PEDOT/PSI films with a dotted line representing convection. (B) Peak current density from entrapment films. (C) Peak current density from dropcast films. (D) Peak current density for films that combine entrapment and drop casting.

Following thorough characterizations of the PEDOT films, PSI was incorporated to determine if there were significant differences in photoactivity across both anion syntheses and differing incorporation methods. As shown in Figure 4 a spread of photoactivities was observed. In Panel A the benefit of PSI incorporation is demonstrated by the sharp increase in photocurrent as compared to a PEDOT film without any PSI entrapment. The Cotrellian diffusion limit can be mitigated by addition of convection to the system through stirring. Panels B, C, and D showcase the peak photocurrents of the films across the three PSI incorporation methods, entrapment, deposition, and the combination of these two, respectively. The PEDOT control films alone were somewhat photoactive with a current density of up to ∼3 μA/cm^2^, with the most pronounced performance being observed when PSI was incorporated into the PEDOT:Br films. The films polymerized in the presence of other anions did not produce comparable results, potentially due to the variety of differing properties that they exhibited. In other words, Photosystem I did not interface as favorably with the other films as it did with PEDOT:Br. Among the three incorporation strategies, entrapment exhibited the lowest photoactivity, which is attributed to the low incorporation of PSI because of diffusion limitations.^14^ The vacuum deposition method increased the current output. A drawback to this approach was that adding a layer of insulative protein atop an electrode significantly reduces the conductivity of the system as shown in SI6. Finally, the combination approach produced the greatest current output of the three. Here, the amount of deposited PSI was maximized but the overall conductivity of the film is reduced.

## Conclusions

The counteranions present during the synthesis of PEDOT have been shown to be of great importance in determining the resulting films properties. These properties have been explored through cyclic voltammetry, electrochemical impedance spectroscopy, and scanning electron microscopy. Through these characterizations, the porous architecture in PEDOT: Br allowed for the greatest ion movement and subsequent charge conduction, consistent with the much greater photoactivity in PEDOT:Br/PSI composite films. These unusual characteristics that PEDOT:Br exhibited are attributed to the oxidation of the bromide electrolyte and formation of bromine during the electrodeposition of EDOT. This work not only contributes to PSI-based biophotovoltaics but also provides fundamental insight on the processing of conductive polymers for optimal performance.

## Supporting Information Available

UV–vis, FTIR, SEM of PSI/PEDOT Films, SEM feature sizes histogram, electropolymerization i-t curves, EIS of PSI atop PEDOT film, profilometry (PDF)

## Abbreviations

PSI: photosystem I
PSII: photosystem II
PEDOT: poly(3,4-ethylenedioxythiophene)
CV: cyclic voltammetry
EIS: electrochemical impedance spectroscopy
SEM: scanning electron microscopy
PCA: photochronoamperometry
OCP: open circuit potential
ITO: indium tin oxide
XPS: X-ray photoelectron spectroscopy
