# Supplementary material for: Counteranion Effects on the Incorporation of Photosystem I with Poly(3,4-ethylenedioxythiophene) (PEDOT)
Source: ACS Omega. 2025 Mar 7;10(10):10199–204. doi: 10.1021/acsomega.4c09448 (PMC11923635; doi:10.1021/acsomega.4c09448)
Supplement: Supplementary file 1 — ao4c09448_si_001.pdf [file ao4c09448_si_001.pdf]

Counter Anion Effects on the Incorporation of Photosystem I with Poly(3,4-ethylenedioxythiophene) (PEDOT)

*William R. Lowery[1], G. Kane Jennings[2], and David E. Cliffel[1]**

William R. Lowery – Department of Chemistry, Vanderbilt University, Tennessee 37235-1822, United States; https://orcid.org/0000-0002-3063-881X

G. Kane Jennings – Department of Chemical and Biomolecular Engineering, Vanderbilt University, Tennessee 37235-1604, United States; https://orcid.org/0000-0002-3531-7388 ;

David E. Cliffel - Department of Chemistry, Vanderbilt University, Tennessee 37235-1822, United States; https://orcid.org/0000-0001-8756-106X; Email: d.cliffel@vanderbilt.edu

Supporting Information

Both UV-Vis and FTIR spectroscopy were utilized to further support that active PSI was incorporated into the various Poly(3,4-ethylenedioxythiophene) (PEDOT)films. An Aglent Technologies Cary 5000 UV-VIS-NIR was utilized to collected absorbance spectrums of the films. These spectra were obtained from the films still directly adhered to the original ITO synthesis substrate. FTIR was also performed directly on the films with use of a Thermo Scientific Nicolet iS5 with an iD7 ATR accessory to allow for direct measurement of the material without any sample preparation post synthesis.

UV-Vis spectra obtained of the entrapment films to support PSI incorporation due to the appearance of peaks at 420 nm and 670 nm compared to control PEDOT films. Both signals are consistent with isolated PSI [1, 2]

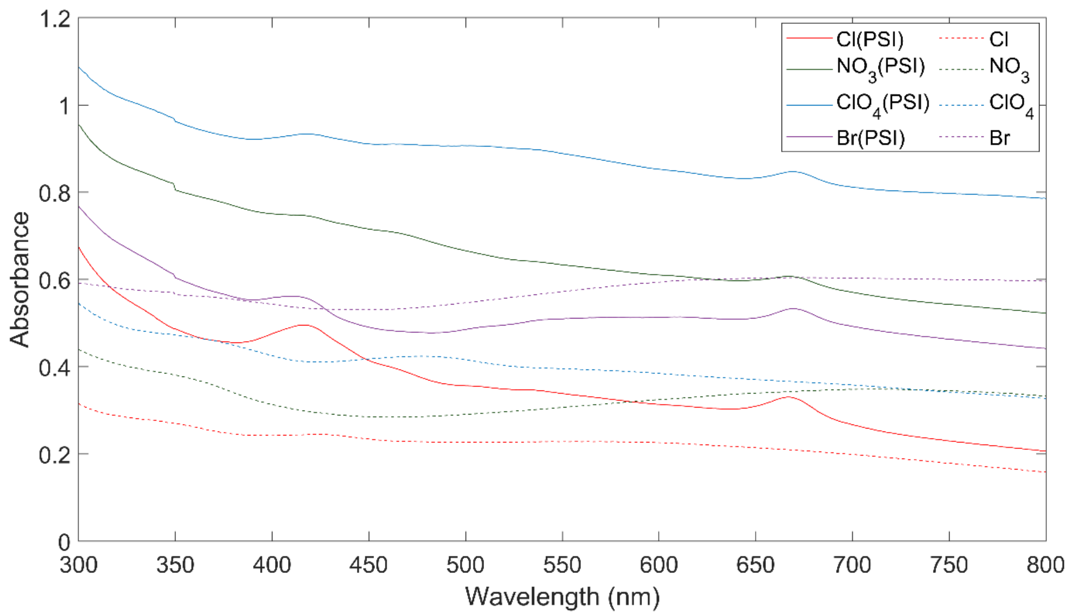

Figure SI1: UV-Vis spectra of PEDOT films. Films polymerized in the presence of PSI all display unique peaks at 420 nm and 670 nm.

FTIR spectra of the different PSI incorporation strategies were obtained to confirm that PSI signatures were apparent along with the above UV-Vis. The amide stretch at 1660 cm^{-1} which has been used extensively to support PSI presence are apparent in all but the entrapment scheme. This is attributable to the minimal incorporation expected from this strategy.

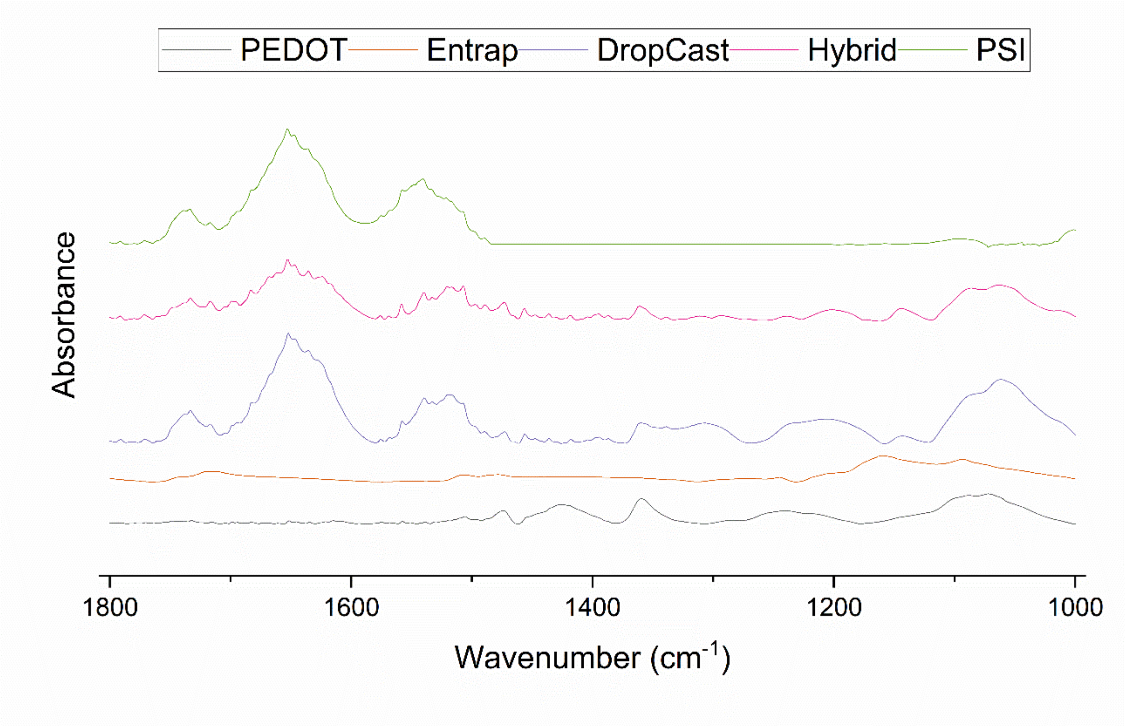

Figure SI2: FTIR spectra of PEDOT films showcasing amide I & II stretches at 1660 cm^{-1} and 1520 cm^{-1} across the various PSI incorporation strategies.

Further SEM comparisons revealed no significant changes to the film morphology by incorporating PSI. The micrographs presented below maintain similar features presented in the main text. PEDOT/PSI films polymerized in the presence of bromide continued to exhibit porous leaf like structures while chloride, perchlorate, and nitrate syntheses yielded globular structures.

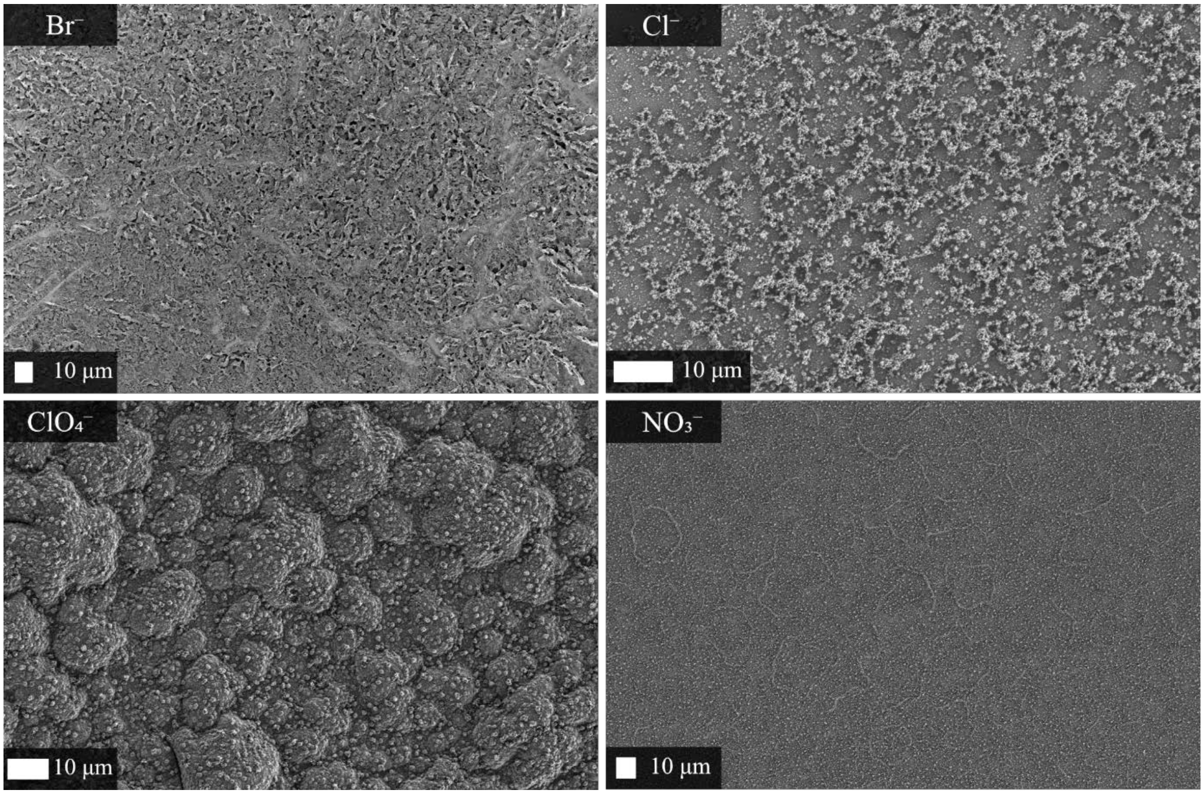

Figure SI3: Scanning electron micrographs of PEDOT films polymerized in the presence of various potassium-based salts along with Photosystem I leading to composite films with interesting morphological changes.

The microstructures observed in **Figure 1** of the main text were analyzed with the assistance of ImageJ to determine feature size.[3] Films polymerized in the presence of chloride exhibited the smallest globule, supporting the conclusion that PEDOT did not grow optimally in the environment. Films polymerized in both nitrate and perchlorate had much higher proliferation leading to larger globule sizes. And finally, the pore sizes of PEDOT:Br films were also analyzed. These pores averaged in size at 210 nm across.

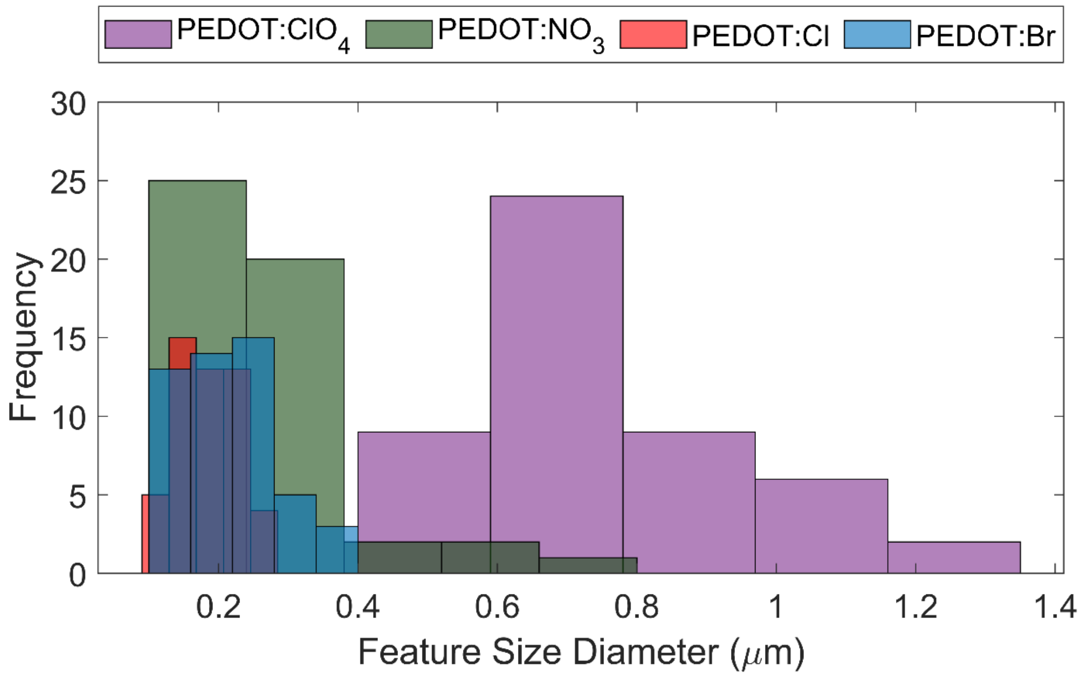

Figure SI4: Histogram plot showcasing feature sizes (globule and pore sizes) of PEDOT films from **Figure 1.**

The 1.6 V potential utilized for electropolymerization of EDOT could oxidize bromide into elemental bromine, as that process occurs at 890 mV.[4, 5] This presence of bromine is indicated by the brown color that is visible in the beaker after a 400 s synthesis cycle. **Figure SI5** shows a picture demonstrating visible bromine along with a close-up of the patterned film of PEDOT:Br. The oxidation of the bromide ion into elemental bromine also provides an explanation for the non-diffusion limited electropolymerization current observed when depositing PEDOT:Br films. When bromide is oxidized to bromine, the recently formed bromine could then further react with the bromide electrolyte to form tribromide. The elemental bromine or tribromide likely blocks further deposition of EDOT forcing a porous structure to be established in the film. As evidence of this mechanism, bromine has been showed to influence the deposition of a similar material, polypyrrole.[6]

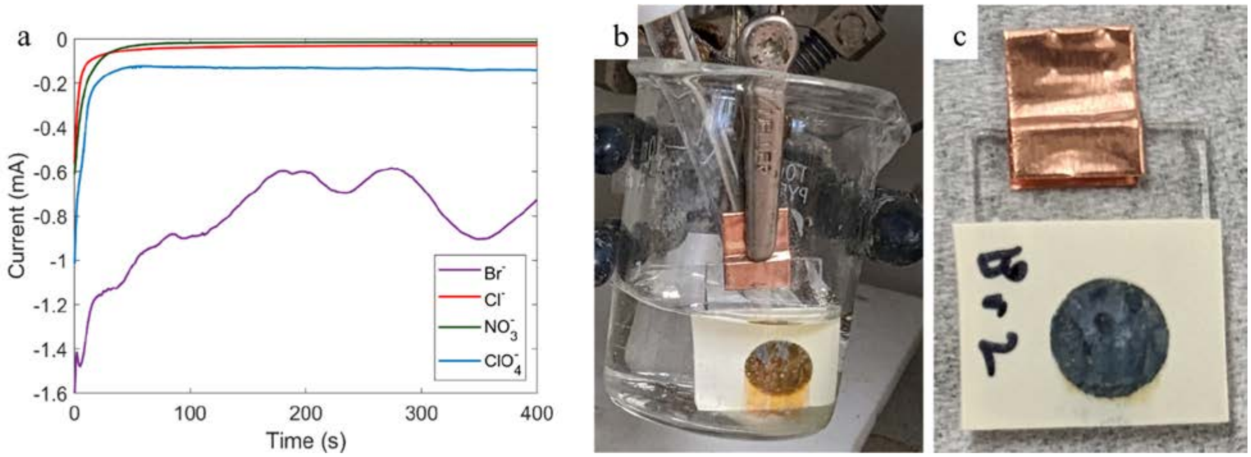

Figure SI5: (a) Electropolymerization i-t curves showcasing the diffusion limited current of EDOT deposition. A non-diffusion limited current was observed when EDOT was deposited in the prescence of KBr due to the high concentration of available bromide for oxidation. (b) Brownish liquid indicative of bromine formation post EDOT deposition. (c) Image of PEDOT:Br film.

Electrochemical Impedance Spectroscopy was performed on bare PEDOT film polymerized on ITO along with polymerized PEDOT films with a drop cast multilayer of PSI to determine the drop in conductivity by inclusion of an insulative protein layer. Experimental parameters were identical to other EIS experiments except for a 2 mM ferri/ferro cyanide redox couple in solution along with a bias potential of 338 mV. The increase in semicircle size is consistent with a larger barrier for electron transfer from the electrode supporting the conclusion that addition of a PSI multilayer would hinder the overall conductivity of the film.

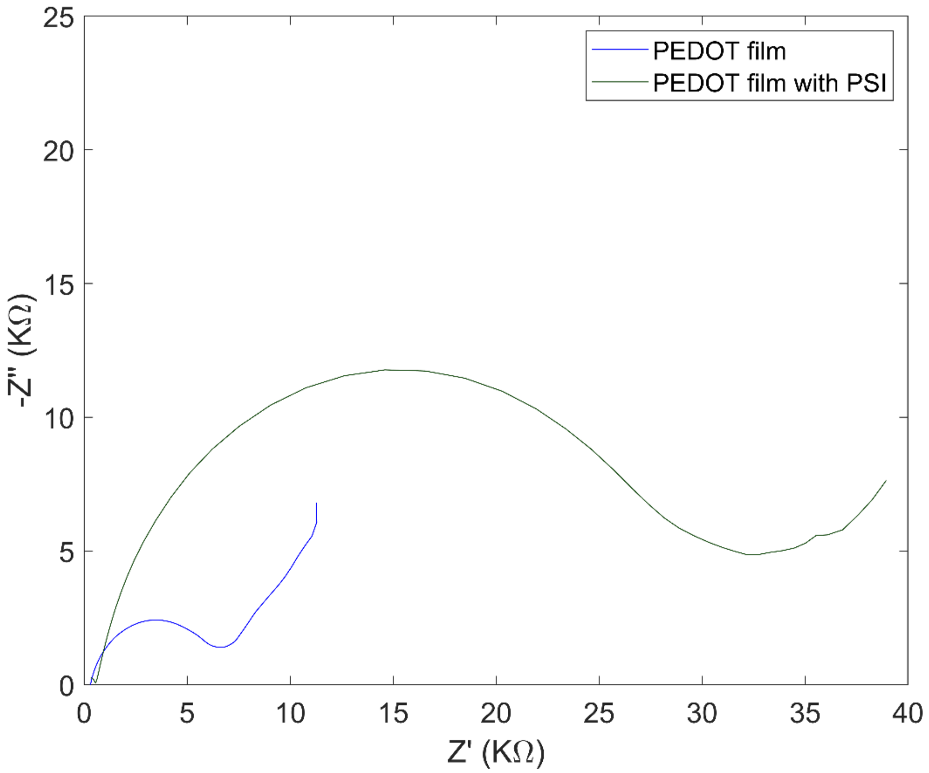

Figure SI6: Electrochemical impedance spectroscopy showcasing the drastic drop in electron transfer efficiency upon introduction of a multilayer of PSI atop a PEDOT film.

Profilometry data was collected utilizing a Bruker Dektak 150. Average surface roughness was calculated relative to the underlying ITO substrate. The data presented below demonstrates how little effect the counter anion has on film thickness. The measurements of perchlorate films should be considered unreliable as the films flaked off readily during measurements and were not fully adhered to the underlying substrate like the other films were.

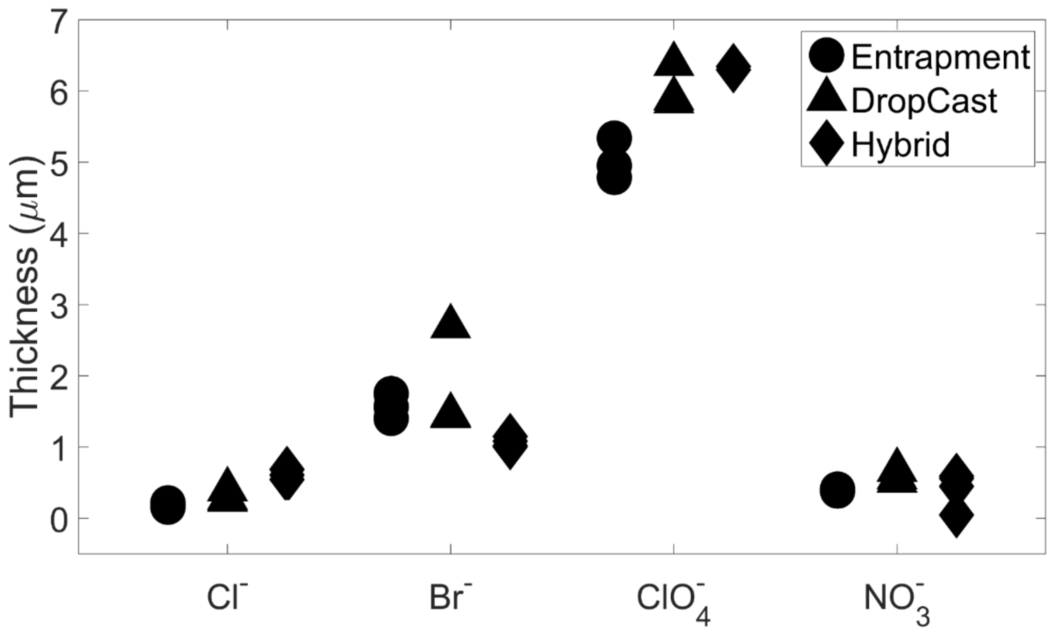

Figure SI7: Profilometry data showcasing film thicknesses of PEDOT films. As expected, the poorly adhered perchlorate films have the largest measurement, whereas the other films show reasonable consistency.

References:

(1) Amjadian, S.; Esmaeilzadeh, M.; Ajeian, R.; Riazi, G.; Ramakrishna, S. Absorption analysis of photosystem I in different plants for bio-hybrid solar cell application. *Energy Technology* **2022**.
(2) Baba, K.; Itoh, S.; Hastings, G.; Hoshina, S. Photoinhibition of Photosystem I electron transfer activity in isolated Photosystem I preparations with different chlorophyll contents. *Photosynthesis Research* **1996**, *47* (2), 121-130.
(3) Schneider, C. A.; Rasband, W. S.; Eliceiri, K. W. NIH Image to ImageJ: 25 years of image analysis. *Nature Methods* **2012**, *9* (7), 671-675.
(4) Bard, A. J.; Faulkner, L. R.; White, H. S. *Electrochemical methods: fundamentals and applications*,Third edition; John Wiley & Sons, Ltd., 2022.
(5) Yoneyama, H.; Laitinen, H. A. Oxidation of bromide ion on tin oxide electrodes. *Journal of Electroanalytical Chemistry and Interfacial Electrochemistry* **1977**, *75* (2), 647-663.
(6) Beck, F.; Oberst, M. Electrocatalytic deposition of polypyrrole in the presence of bromide. *Journal of Applied Electrochemistry* **1992**, *22* (4), 332-340.
